# Effect of overliming and activated carbon detoxification on inhibitors removal and butanol fermentation of poplar prehydrolysates

**DOI:** 10.1186/s13068-018-1182-0

**Published:** 2018-06-26

**Authors:** Yu Zhang, Changlei Xia, Mingming Lu, Maobing Tu

**Affiliations:** Department of Chemical and Environmental Engineering, University of Cincinnati, 2901 Woodside Drive, Cincinnati, OH 45221 USA

**Keywords:** Prehydrolysates, Carbonyl inhibitors, Detoxification, Acetone–butanol–ethanol (ABE) fermentation

## Abstract

**Background:**

Biomass prehydrolysates from dilute acid pretreatment contain a considerable amount of fermentable sugars for biofuels production. However, carbonyl degradation compounds present severe toxicity to fermentation microbes. Furans (such as furfural and hydroxymethylfurfural), aliphatic acids (such as acetic acid, formic acid and levulinic acid) and phenolic compounds (such as vanillin and syringaldehyde) have been suggested to be the main inhibitors in biomass prehydrolysates. However, no single compound has been determined as the dominant toxic inhibitor. The effects of various detoxification methods on inhibitors removal have not been fully understood.

**Results:**

The effects of overliming and activated carbon (AC) detoxification on the removal of inhibitors and butanol fermentation of the poplar prehydrolysates were investigated. Gas chromatography–mass spectrometry (GC/MS) was used to identify and quantify 46 carbonyl compounds as potential inhibitors. It was observed that overliming and AC treatment alone did not make the prehydrolysates fermentable with *Clostridium saccharobutylicum*. The sequential overliming and AC resulted in a remarkable fermentability and a high butanol yield at 0.22 g g^−1^ sugar. The inhibitor removal in the prehydrolysates treated by overliming and AC was also examined by GC/MS. Overliming removed 75.6% of furan derivatives and 68.1% of aromatic monomers. In comparison, AC (5.0% *w/v*) removed 77.9% of furan derivatives and 98.6% of aromatic monomers. In addition, overliming removed much more 2,5-furandicarboxyaldehyde, 5-ethylfuran-2-carbaldehyde and 2,5-hexanedione than AC did. On the contrary, AC could remove considerably more phenolic acids than overliming. In the sequential detoxification, both dialdehydes/diketones and phenolic acids were extensively removed. This could be the main reason why the sequential detoxification enabled a remarkable ABE fermentation for the prehydrolysates.

**Conclusions:**

This study indicated that the effect of overliming and AC treatment on inhibitors removal was related to their chemical structures. Overliming removed more dialdehydes and diketones than AC treatment, while AC removed more phenolic acids than overliming. Sequential overliming and AC treatment were required to make the prehydrolysates fermentable with *C. saccharobutylicum*. The study also suggested different detoxification method was needed for ABE fermentation of the prehydrolysate as compared to ethanol fermentation.

**Electronic supplementary material:**

The online version of this article (10.1186/s13068-018-1182-0) contains supplementary material, which is available to authorized users.

## Background

Lignocellulosic biomass has the great potential to be used for producing biofuels sustainably [1]. To break down the biomass recalcitrance, diluted acid is typically used to pretreat biomass and enhance cellulose accessibility for subsequent enzymatic hydrolysis [2, 3]. Hemicellulose can be hydrolyzed to monomeric sugars in the prehydrolysates after dilute acid pretreatment and is available for further biofuels production [4]. However, considerable amount of inhibitors have been generated in dilute acid pretreatment, which hinder microbial fermentation [5–9]. Biomass-derived inhibitors include furfural, hydroxymethylfurfural (HMF), aliphatic acids and phenolic compounds [10, 11]. Several studies have been concentrated on the identification of potential inhibitors [12, 13]. Chen et al. developed a HPLC method to quantify 32 aliphatic acids, aromatic acids, aldehydes and phenolic compounds in a corn stover hydrolysate [14]. Sharma et al. characterized 40 potential acid inhibitors in biomass prehydrolysates with HPLC–MS/MS [12]. Klinke et al. reported 26 degradation products resulted from alkaline treated wheat straw hydrolysates, including acids, furans and phenols [13]. Although significant efforts have been made to identify the potential inhibitors on microbial fermentation, no single compound has been determined to be the dominant inhibitor [15]. Furfural and HMF content has been suggested to be the important indicator of prehydrolysate toxicity, but they are not the major inhibitors [4, 16]. Aromatic alcohols (catechol and coniferyl alcohol) and aromatic aldehydes (4-hydroxybenzaldehyde and syringaldehyde) were also found to inhibit the growth and fermentation of *Escherichia coli* LY01 [17, 18], and their toxicity was directly related to the hydrophobicity [19]. Indeed, aldehydes and ketones are generally considered as major detrimental compounds to the microorganisms. For instance, Ando et al. quantified 12 aromatic degradation compounds in the poplar hydrolysates and also investigated the influences on yeast fermentation [20]. The results indicated that the aldehydes and ketones were more inhibitory than the corresponding acids and alcohols. Therefore, the identification and detoxification of aldehydes and ketones in the prehydrolysates are critically needed in biofuels fermentation.

Several methods have been proposed to alleviate the negative effects of the inhibitors in biomass hydrolysates or prehydrolysates, including overliming [21], anion exchange resin treatment [22], activated carbon (AC) [23, 24], sulfite treatment [25], and treatment with laccase and fungi [25]. Overliming has been suggested to be one of the most effective detoxification methods [26, 27]. Alkaline conditions facilitated the aldol reactions between aldehydes and ketones, and the oxidation of carbonyl compounds could potentially mitigate their toxicity [28–30]. Martin et al. observed that overliming partially reduced the concentration of furfural, HMF and phenolic compounds, resulting in an increased ethanol yield from 0.38 to 0.52 g g^−1^ glucose in the yeast fermentation [31]. Although overliming facilitated the yeast fermentation, acetone–butanol–ethanol (ABE) fermentation was still limited due to the remaining inhibitory compounds and high sensitivity of *Clostridium* [32].

Compared to the chemical detoxification methods, AC removed inhibitors by physical adsorption [33]. Gong et al. reported that AC provided a comparable productivity (0.40 g L^−1^ h^−1^) in the yeast fermentation of sugarcane bagasse hydrolysates, compared with pH adjustment and ion-exchange resins [34]. Lu et al. found that AC significantly removed most of the furans and phenolic compounds in the hydrolysates, but the butanol production could only be improved to 59.2% of the control [32]. Wang et al. achieved a 20% improvement of xylitol yield after AC detoxification [35]. Villarreal et al. conducted fermentation with the prehydrolysate detoxified by AC; however, the highest productivity only reached 37% of the reference fermentation [36]. In our preliminary study, it was observed that overliming and AC treatment alone could not make the prehydrolysates fermentable with *Clostridium saccharobutylicum*. However, the prehydrolysate treated with a sequential overliming and AC method was fermentable.

In this study, GC/MS without derivatization was used to analyze the composition changes in the poplar prehydrolysates after overliming and AC detoxification. The ABE fermentability of the detoxified prehydrolysates was examined using *C. saccharobutylicum.* Liquid–liquid extraction with dichloromethane (DCM) was used to isolate the potential inhibitors from the prehydrolysates. It is hypothesized that overliming and AC treatment not only removed some common inhibitors, but also selectively eliminated some specific inhibitors. It is also hypothesized that AC is more effective in removing phenolic acids due to their hydrophobicity and overliming is more selective in removing certain dialdehydes and diketones due to the base-catalyzed aldol-condensation reactions. As a result, a combination approach is needed to detoxify the prehydrolysates for ABE fermentation. Fermentability of the detoxified prehydrolysates was also compared between yeast and *C. saccharobutylicum*. This study is expected to further advance the understanding of the potential inhibitors in the biomass prehydrolysates and the detoxification mechanism by overliming.

## Results and discussion

### Effects of overliming and activated carbon on inhibitors removal

**Fig. 1:**
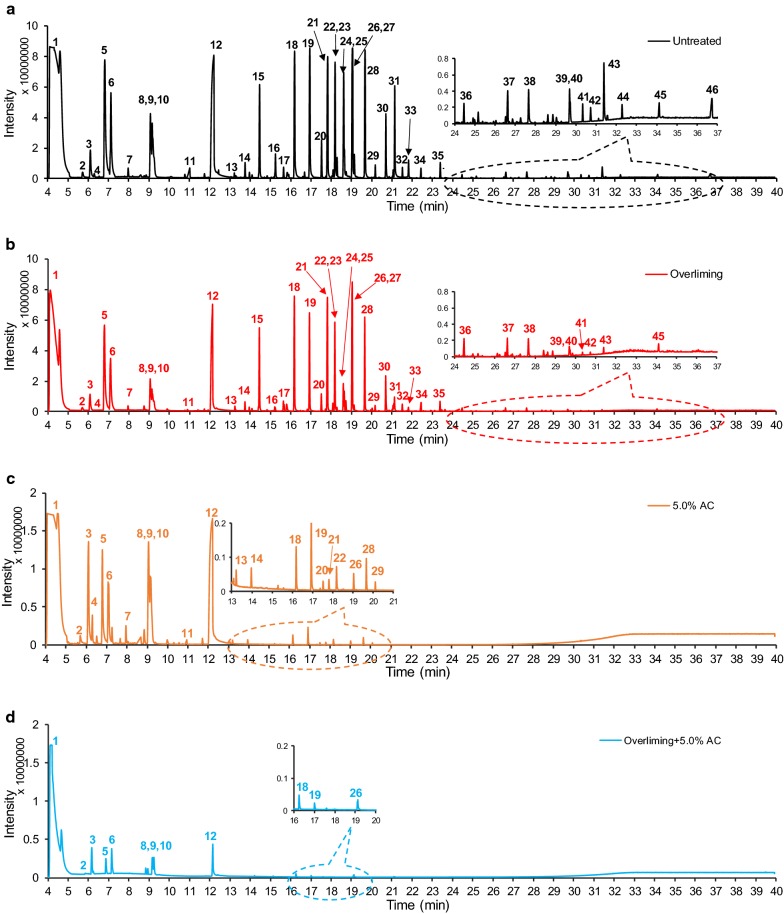
TIC-GC/MS chromatograms of the prehydrolysates treated with different detoxification methods. **a** Untreated prehydrolysates, **b** overliming; **c** 5% AC; and **d** sequential overliming and 5% AC

The potential inhibitors extracted from the poplar prehydrolysates were determined by GC/MS. The corresponding 46 inhibitory compounds (Fig. 1a) from untreated prehydrolysates were identified together with their retention time (RT), mass to charge ratio (*m/z*), and concentrations (Table 1). The inhibitory compounds can be divided into three groups. The first group (RT: 4–13 min) are furan derivatives (such as furfural, 2,5-furandicarboxyaldehyde, and HMF) and aliphatic derivatives (2,5-hexanedione and 3-hexen-2-one). 2,5-furandicarboxyaldehyde and 2,5-hexanedione, as a dialdehyde and a diketone, were first reported in the biomass prehydrolysates. The second group (RT: 13–24 min) are aromatic monomers (such as vanillin, syringaldehyde, and (4-hydroxy-3,5-dimethoxybenzoyl)-acetaldehyde). The third group (RT: 24–37 min) are aromatic dimers (such as 4-hydroxyphenyl 3,4,5-trimethoxybenzoate and syringil) and their concentrations are significantly less than the first and second groups of compounds (1–10%). Their corresponding chemical structures and fragment patterns are shown in Additional file 1: Figs. S1–S46.

**Table 1:** Effect of overliming and AC detoxification on inhibitors removal

GC peak	Compound name	RT^a^ (min)	*m/z*	Inhibitor concentration^b^ (mg L^−1^)				
				Untreated	Overliming	5.0% AC	Overliming + 2.5% AC	Overliming + 5.0% AC
	*Furan derivatives*			4444.3	1078.6	995.1	897.3	408.7
1*	Furfural	4.089	96	3360.9	736.8	721.8	789.3	378.4
2*	2-Acetylfuran	5.706	110	3.3	0.9	0.9	0.9	0.8
5*	5-Methylfurfural	6.794	110	156.3	27.5	16.8	25.3	1.9
7*	Cyclotene	7.965	112	3.8	1.0	1.7	0.7	NA
8**	2,5-Furandicarboxyaldehyde	9.063	124	148.8^c^	29.9^c^	53.6^c^	11.6^c^	7.1^c^
9**	2-Furyl hydroxymethyl ketone	9.15	126	88.4^c^	59.5^c^	20.3^c^	34.1^c^	5.9^c^
10*	5-Ethylfuran-2-carbaldehyde	9.226	124	57.3	6.2	17.6	4.4	3.1
12*	Hydroxymethylfurfural	12.199	126	625.5	216.8	162.4	31.0	11.6
	*Aliphatic derivatives*			26.8	11.2	22.3	7.2	5.2
3*	2,5-Hexanedione	6.094	114	24.9	10.4	20.5	7.2	5.2
4**	3-Hexen-2-one	6.286	98	1.9^d^	0.8^d^	1.9^d^	NA	NA
	*Aromatic monomers*			1198.8	383.5	17.0	48.2	4.5
6*	Phenol	7.109	94	68.2	16.7	5.3	5.1	2.7
11*	Benzoic acid	11.007	122	74.9	3.8	4.7	0.4	NA
13*	3′-Methoxyacetophenone	13.211	150	5.6	2.1	NA	0.8	NA
14*	3,4,5-Trihydroxybenzaldehyde	13.74	154	6.4	1.7	0.4	0.2	NA
15*	Vanillin	14.458	152	72.8	25.8	NA	NA	NA
16***	Homovanillin	15.263	166	17.0^e^	1.9^e^	NA	NA	NA
17*	Acetovanillone	15.636	166	10.3	9.2	NA	0.1	NA
18*	Guaiacylacetone	16.188	180	205.5	73.5	2.0	12.8	0.9
19**	1-(4-Hydroxy-3-methoxyphenyl)propane-1,2-dione	16.942	194	121.0^e^	20.3^e^	2.1^e^	4.9^e^	0.2^e^
20***	1-(3,4,5-Trihydroxyphenyl)propane-1,2-dione	17.522	196	23.1^e^	9.8^e^	0.2^e^	1.0^e^	NA
21*	Syringaldehyde	17.826	182	110.0	36.3	0.3	1.7	NA
22***	Hydroxypropiovanillone	18.189	196	83.4^e^	41.2^e^	0.7^e^	3.5^e^	NA
23**	Homosyringaldehyde	18.29	196	9.7^f^	0.9^f^	NA	0.1^f^	NA
24**	1-Hydroxy-3-(4-hydroxy-3-methoxyphenyl)propan-2-one	18.617	196	0.7^e^	NA	NA	NA	NA
25*	Acetosyringone	18.653	196	57.8	27.0	NA	0.2	NA
26*	Syringylacetone	19.055	210	88.5	29.7	0.3	9.5	0.7
27*	1-(4-Hydroxy-3-methoxyphenyl)-2-butanone	19.142	194	11.3^e^	2.1^e^	NA	NA	NA
28***	1-(4-Hydroxy-3,5-dimethoxyphenyl)propane-1,2-dione	19.672	224	104.8^f^	15.4^f^	0.8^f^	5.7^f^	NA
29***	1-Hydroxy-1-(4-hydroxy-3,5-dimethoxyphenyl)-2-propanone	20.179	226	6.7^f^	4.4^f^	0.2^f^	0.3^f^	NA
30***	2-Hydroxy-1-(4-hydroxy-3,5-dimethoxyphenyl)propan-1-one	20.701	226	37.5^f^	29.2^f^	NA	1.5^f^	NA
31***	2-Hydroxy-1-syringyl-ethanone	21.14	226	59.7^f^	23.7^f^	NA	0.4^f^	NA
32***	1-(3,4,5-Trimethoxyphenyl)-1,2-propanedione	21.517	238	4.9^f^	2.8^f^	NA	NA	NA
33***	(4-Hydroxy-3,5-dimethoxybenzoyl)-acetaldehyde	21.822	224	9.5^f^	0.4^f^	NA	NA	NA
35***	1-(4-Hydroxy-3,5-dimethoxyphenyl)pentane-1,2-dione	23.388	252	9.5^f^	5.4^f^	NA	NA	NA
	*Aromatic dimers*			45.9	14.2	NA	NA	NA
34***	Gentisein	22.333	244	5.6^f^	4.0^f^	NA	NA	NA
36***	2-(4-Hydroxy-3-methoxyphenyl)-1-(3,5-dihydroxyphenyl)ethanone	24.465	274	2.4^f^	1.9^f^	NA	NA	NA
37***	4-Hydroxyphenyl 3,4,5-trimethoxybenzoate	26.633	304	4.8^f^	2.1^f^	NA	NA	NA
38***	1,2-Bis(4-Hydroxy-3-methoxyphenyl)ethanone	27.666	288	5.3^f^	2.6^f^	NA	NA	NA
39***	1-(4-Hydroxy-3,5-dimethoxyphenyl)-2-(4-hydroxy-3-methoxyphenyl)ethanon	29.714	318	4.5^f^	1.1^f^	NA	NA	NA
40***	2-(4-Hydroxy-3,5-dimethoxyphenyl)-1-(4-hydroxy-3-methoxyphenyl)ethanone	29.725	318	2.2^f^	0.6^f^	NA	NA	NA
41***	2-Syringylacetosyringone	30.345	362	2.0^f^	0.2^f^	NA	NA	NA
42***	Vanillosyringil	30.748	332	1.8^f^	0.2^f^	NA	NA	NA
43***	1,2-bis(4-Hydroxy-3,5-dimethoxyphenyl)ethanone	31.397	348	8.4^f^	0.6^f^	NA	NA	NA
44***	Syringil	32.299	362	1.7^f^	NA	NA	NA	NA
45***	1-(4-Acetyl-3,5-dimethoxyphenyl)-2-(4-hydroxy-3,5-dimethoxyphenyl)ethane-1,2-dione	34.138	388	2.3^f^	0.9^f^	NA	NA	NA
46***	Phenol, 4,4′-(1,2-ethanediyl)bis[2,6-dimethoxy-,diacetate	36.744	418	4.9^f^	NA	NA	NA	NA

* The compounds verified with standard

** The compounds compared with reference mass spectrum

*** The compounds derived by the fragments

^a^RT are shortened for the retention time

^b^Inhibitor residual was calculated based on the intergradation area of each compound

^c^The concentration was determined by the calibration of hydroxymethylfurfural

^d^The concentration was determined by the calibration of 2,5-hexanedione

^e^The concentration was determined by the calibration of vanillin

^f^The concentration was determined by the calibration of syringaldehyde

Both overliming and AC significantly removed the identified compounds, especially for aromatic monomers and dimers (Fig. 1). AC treatment appeared to remove a higher percentage of most compounds than overliming except 2,5-furandicarboxyaldehyde, 5-ethylfuran-2-carbaldehyde and 2,5-hexanedione. The sequential overliming and AC removed most of the first, second and third group compounds (Fig. 1d). Only the sequential approach enabled a dramatic improvement on ABE fermentation of the poplar prehydrolysates in this study.

Specifically for overliming treatment, furfural content was reduced by 78%, from 3360.9 to 736.8 mg L^−1^ after overliming treatment (determined by GC/MS, Table 1). HMF content was reduced by 65%, from 625.5 to 216.8 mg L^−1^ (Table 1). Similarly for AC treatment, furfural content was reduced by 79% and HMF content was reduced by 74% (determined by GC/MS). Previously, similar results have been reported on the bagasse hydrolysates detoxification by Ca(OH)_2_ [26]. Sequential treatment with overliming and 5.0% AC could further reduce the furfural and HMF content to 378.4 and 11.6 mg L^−1^, respectively (determined by GC/MS). Although furfural and HMF were not the strong inhibitors, they have been suggested to be important indicators for relative toxicity of prehydrolysates [4].

As for the aromatic monomers, overliming removed vanillin, syringaldehyde and acetosyringone by 65, 67 and 53%, respectively. However, 5.0% AC and the sequential approach completely removed all three compounds. This indicated that AC was more effective in removing aromatic monomers than overliming. As for the aromatic dimers, overliming reduced gentisein by 29% (from 5.6 to 4.0 mg L^−1^), 4-hydroxyphenyl 3,4,5-trimethoxybenzoate by 56% (from 4.8 to 2.1 mg L^−1^), while 5.0% AC removed all 12 aromatic dimers completely. This suggested AC was more efficient in removing aromatic dimers due to their higher hydrophobicity.

**Fig. 2:**
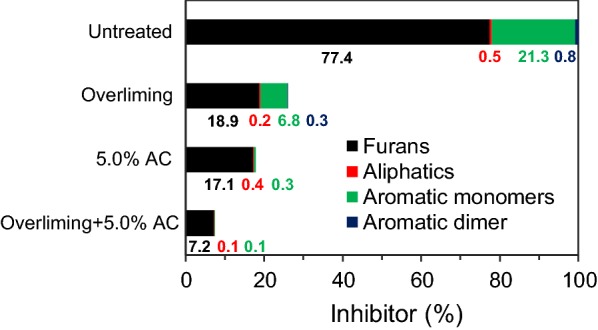
Inhibitors composition change of the detoxified prehydrolysates. The inhibitor concentration in the untreated prehydrolysates was assumed as 100%. The percentage were calculated based on the mass concentration in Table 1

As summarized in Fig. 2, the potential inhibitors from the untreated prehydrolysates are composed of 77.4% furans, 21.3% aromatic monomers, and a minor number of aliphatic derivatives and aromatic dimers. The inhibitory effects of the degradation compounds vary considerably according to their chemical structures [6]. Low concentration (< 3 g L^−1^) of furfural and HMF was reported to increase ABE yield with *C. beijerinckii* BA101 [37]. This stimulatory effect could be resulted from the change of redox balance in *C. beijerinckii*, because NADPH or NADH was used to reduce furfural to furfural alcohol [38]. Aromatic aldehydes (such as benzaldehyde, 2-hydroxybenzaldehyde and vanillin) has been reported to limit the growth and butanol yield of *C. acetobutylicum* [39].

The results showed overliming removed 75.6% of furan derivatives and 68.1% of aromatic monomers (Fig. 2). Base-catalyzed aldol condensations have been suggested to be the main reactions in removing furan aldehydes previously [21]. The same reaction could occur for 2,5-hexanedione. In comparison, 5.0% AC removed 77.9% of furan derivatives and 98.6% of aromatic monomers. More aromatic monomers and dimers have been removed by AC than those by overliming. It indicated AC was more effective in removing aromatic compounds from the prehydrolysates. Similar results have been reported before [40], in which 38.7% of furfural and 57.5% of total phenolic compounds were reduced by AC and 45.8% of furans and 35.8% of the phenolics were eliminated by overliming. Previously, it has been suggested the AC can selectively remove furans and phenolic compounds other than carboxylic acids due to its strong hydrophobicity [41]. Sequential overliming and 5.0% AC removed the 90.7% of furans and 99.5% of aromatic monomers (Fig. 2). Previously, Ultraviolet–Visible (UV–Vis) spectra have been used to monitor furans in the prehydrolysates [42]. In this study, a major peak at 278 nm was observed in the untreated and treated prehydrolysates (Additional file 1: Fig. S48). The absorbance at 278 nm dropped by 12.6 and 77.0% after overliming and 5.0% AC detoxification, respectively. It indicated that absorbance at 278 nm was not only related to furans, but also associated with phenolic compounds. As a result, integration of overliming and AC treatment could be more effective to remove both furans and phenolic compounds (aromatic monomers and dimers). Its effectiveness will be assessed in the following ABE fermentation processes.

As for organic acid removal, without derivatization of the extracted samples, few organic acids were detected by GC/MS in this study. Previously, more than 30 aliphatic and phenolic acids have been identified in a poplar hydrolysate after trifluorobis(trimethylsilyl)acetamide and chlorotrimethylsilane derivatization [43]. In this research, HPLC and LC-QTOF were used to determine the short chain aliphatic acids and phenolic acids removal after detoxification (Table 2). It was observed that aliphatic acids were not changed much with overliming and AC treatment, even the sequential overliming and AC treatment only removed 17% of formic acid and 10% levulinic acid. However, the overliming and AC showed significant difference in phenolic acids removal. AC removed considerably more phenolic acids than overliming. The results showed cinnamic acid, dihydroxybenzoic acid, coumaric acid, vanillic acid and ferulic acid could be removed by 100, 100, 100, 85.74 and 10%, respectively, after AC treatment (Table 2). However, they were reduced only by 74.69, 13.99, 1.13, 32.61 and 60.94%, respectively, after overliming treatment. Consequently, the sequential detoxification removed 96.8% of the total phenolic acids. The difference in removing the dialdehydes/diketones and phenolic acids by overliming and AC could be used to explain their synergic effect in detoxification of the prehydrolysates for ABE fermentation.

**Table 2:** Effect of detoxification on organic acid removal

Compound name	Organic acid removal percentage (%)		
	Overliming	5.0% AC	Overliming + 5.0% AC
*Aliphatic acids*			
Formic acid	8.0	14.0	17.0
Acetic acid	0.0	0.0	0.0
Levulinic acid	5.0	6.0	10.0
*Phenolic acids*			
Benzoic acid	32.2	49.5	68.2
Cinnamic acid	74.7	100.0	100.0
Dihydroxybenzoic acid	14.0	100.0	100.0
Coumaric acid	1.1	100.0	100.0
Vanillic acid	32.6	85.7	93.9
Homovanillic acid	62.1	89.5	100.0
Ferulic acid	60.9	100.0	100.0
Syringylglycolic acid	38.8	100.0	100.0

It should be noted that both overliming and AC detoxification would result in sugar loss. The results showed that 7.2% of sugars were lost in overliming compared to 4.8% of sugars in 5.0% AC. The sequential overliming and 5.0% AC resulted in 8.4% sugar loss (Table 3).

**Table 3:** Effect of detoxification on sugar loss and total inhibitor concentrations in the prehydrolysates

Treatment	Sugar concentration (g L^−1^)					Total inhibitor concentration (g L^−1^)
	Glucose	Xylose	Galactose	Arabinose	Mannose	
Untreated	67.85 ± 1.67	8.93 ± 0.13	1.04 ± 0.10	0.64 ± 0.08	1.94 ± 0.10	5.68
Overliming	62.98 ± 0.31	8.00 ± 0.23	0.98 ± 0.11	0.56 ± 0.11	2.12 ± 0.12	1.47
5.0% AC	64.85 ± 0.38	8.26 ± 0.03	0.93 ± 0.05	0.61 ± 0.05	1.89 ± 0.13	1.00
Overliming + 5.0% AC	62.51 ± 0.28	7.84 ± 0.33	0.93 ± 0.19	0.51 ± 0.05	1.89 ± 0.08	0.41

### Synergistic effect of overliming and AC on ABE fermentation

ABE fermentation with *C. saccharobutylicum* was performed with the prehydrolysates detoxified by overliming, 5.0% AC, and sequential overliming and 5.0% AC (Table 4). The results showed that both overliming and 5.0% AC alone could not make the prehydrolysates fermentable. The sequential overliming and 5.0% AC resulted in remarkable fermentability and high-butanol yield (0.22 g g^−1^ sugar). This agrees well with a recent report on butanol yield by *Clostridium beijerinckii CC101* [44]. The final concentration of acetone, butanol and ethanol was 7.1, 13.2 and 1.0 g L^−1^, respectively. The ABE yield reached 0.35 g g^−1^ sugar, which is similar to the pure glucose fermentation (0.36 g g^−1^ sugar). This indicated that overliming and AC treatment displayed a synergistic effect on detoxification of the prehydrolysates. Overliming and AC detoxified some common inhibitors such as furan derivatives and phenolic aldehydes and ketones, but also selectively removed some specific inhibitors. Table 1 showed overliming removed much more 2,5-furandicarboxyaldehyde, 5-ethylfuran-2-carbaldehyde and 2,5-hexanedione than AC did. For example, 2,5-furandicarboxyaldehyde was reduced from 148.8 to 29.9 mg L^−1^ by overliming and only to 53.6 mg L^−1^ by AC. 2,5-furandicarboxyaldehyde and 2,5-hexanedione are a dialdehyde and a diketone. On the contrary, AC appeared to be much more effective in removing phenolic acids (Table 2). Coumaric acid, vanillic acid and ferulic acid have been reported to inhibit butanol fermentation by *Clostridium beijerinckii* [45]. In the sequential detoxification, both dialdehyde/diketone and phenolic acids were significantly removed (Tables 1, 2). This could be the reason why sequential detoxification enabled a remarkable ABE fermentation for the prehydrolysates. It should be noted that not much aliphatic acids (formic, acetic and levulinic acids) were removed in the sequential detoxification (Table 2).

**Table 4:** Effect of detoxification methods on products yield in ABE fermentation

Sample	Total sugar after fermentation^a^ (g L^−1^)	*C*_acids_ (g L^−1^)	*C*_butanol_ (g L^−1^)	*Y*_butanol_ (g g^−1^ sugar)	*C*_ABE_ (g L^−1^)	*Y*_ABE_ (g g^−1^ sugar)
Glucose control	10.28 ± 1.10	0.68 ± 0.08	11.18 ± 0.03	0.25 ± 0.01	15.94 ± 0.16	0.36 ± 0.02
Untreated	67.19 ± 0.81	6.18 ± 0.21	0.00 ± 0.00	0.00 ± 0.00	0.00 ± 0.00	0.00 ± 0.00
Overliming	62.01 ± 1.75	6.05 ± 0.19	0.00 ± 0.00	0.00 ± 0.00	0.00 ± 0.00	0.00 ± 0.00
5.0% AC	63.70 ± 1.06	6.22 ± 0.17	0.00 ± 0.00	0.00 ± 0.00	0.00 ± 0.00	0.00 ± 0.00
Overliming + 5.0% AC	5.89 ± 0.25	3.93 ± 0.29	13.36 ± 0.35	0.22 ± 0.02	21.26 ± 0.47	0.35 ± 0.09

*C*_acids_, represents the final concentration of the acetic acid and butyric acid by 96 h; *C*_butanol_, represents the butanol production by 96 h; *Y*_butanol_, represents the butanol yield at 96 h based on the total sugar consumption; *C*_ABE_, represents ABE production by 96 h; *Y*_ABE_, represents the ABE yield at 96 h based on the total sugar consumption

^a^Original sugar concentrations of fermentation were diluted to 90% of the prehydrolysates due to the inoculum of *Clostridium*. The initial concentration of glucose control was 54.86 g L^−1^

Previous studies indicated that furans and phenolics were reduced considerably by overliming [4], while formic acid, acetic acid and levulinic acid remained unchanged. Although acetic acid can be consumed by *C. saccharobutylicum,* formic acid and levulinic acid could be toxic to *Clostridium*. A significant amount of acetic acid (~ 8.6 g L^−1^) was presented in the detoxified prehydrolysates, most of which was consumed and converted to acetone (7.0 g L^−1^) after 96 h.

### Effect of activated carbon dosage in sequential detoxification on ABE fermentation

**Fig. 3:**
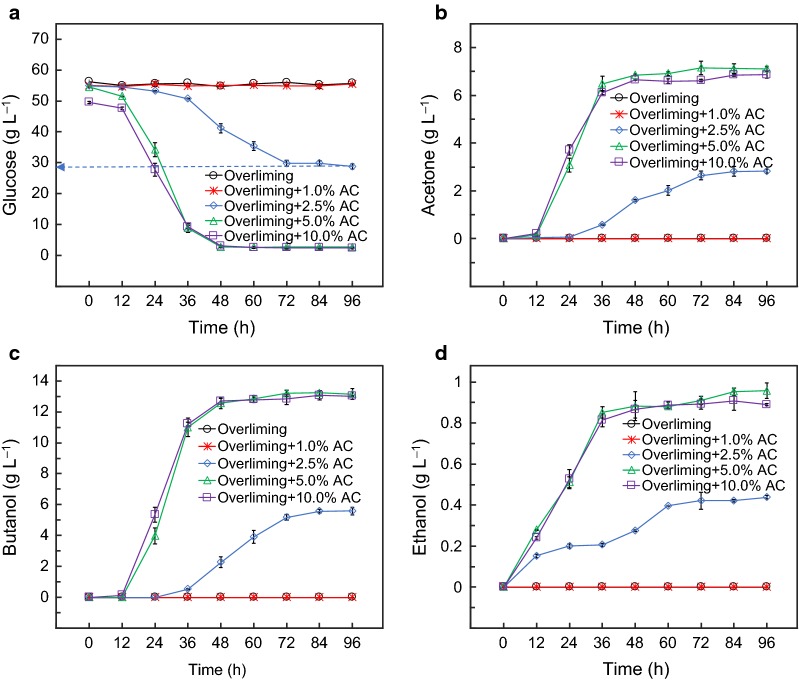
Effect of sequential detoxification on ABE fermentation of the propolar prehydrolysate. **a** glucose consumption, **b** acetone production, **c** ethanol production, and **d** butanol production

AC detoxification efficiency is dependent on how much AC used in the process [23, 46, 47]. In the sequential detoxification process, the prehydrolysates after overliming were further treated with 1.0, 2.5, 5.0 and 10.0% (*w/v*) of AC. The results showed that no ABE was produced with the prehydrolysate treated with sequential overliming and 1.0% AC (Fig. 3). Approximately 5.6 g L^−1^ of butanol and 8.9 g L^−1^ of ABE were produced with the prehydrolysate treated with sequential overliming and 2.5% AC. The final acetone and ethanol concentration was 2.8 and 0.4 g L^−1^, respectively. A considerable amount of glucose (28 g L^−1^) remained after 96 h fermentation. It indicated the prehydrolysate was partially detoxified under this condition. Butanol production reached 13.2 g L^−1^ with the prehydrolysate treated with sequential overliming and 5.0% AC. The ABE production was 21.3 g L^−1^, which were similar to the prehydrolysate treated with sequential overliming and 10.0% AC. It should be noted that sequential overliming and 10.0% AC detoxification resulted in more sugar loss than sequential overliming and 5.0% AC detoxification. Similar observation of dosage dependence has been reported on the formic acid removal by AC [23], in which 1.0% AC removed 47.3% furfural and 5.0% AC removed 75.5% of furfural in a hardwood prehydrolysate.

### Effect of overliming on yeast fermentation

To examine the different tolerance between yeast and *C. saccharobutylicum*, the overliming treated prehydrolysates was fermented with yeast as well (Table 5) [48–50]. The results showed that the overliming detoxified prehydrolysates exhibited fermentability comparable to the glucose control. Specifically, the ethanol yield reached 0.40 g g^−1^ sugar within 12 h. Similar results have been reported by examining the effects of carbonyl inhibitors on the ethanol fermentation [8, 30, 51, 52]. Martiniz et al. obtained an ethanol yield of 0.45 g g^−1^ sugar with *Escherichia coli* after overliming detoxification [26]. As mentioned above, the overliming treated prehydrolysate was not fermentable with *C. saccharobutylicum.* This indicated that *C. saccharobutylicum* is more sensitive to the prehydrolysate inhibitors and a different detoxification approach is required for ABE fermentation with *C. saccharobutylicum*.

**Table 5:** Effect of overliming on the initial sugar concentrations and yeast fermentations

Sample	Initial sugar concentration (g L^−1^)					Total sugar consumption^a^ (g L^−1^)	*C*_ethanol_^b^ (g L^−1^)	*Y*_ethanol_^c^ (g g^−1^ sugar)
	Glucose	Xylose	Galactose	Arabinose	Mannose			
Glucose control	20.11 ± 0.36	0.00 ± 0.00	0.00 ± 0.00	0.00 ± 0.00	0.00 ± 0.00	20.11 ± 0.36	8.32 ± 0.20	0.41 ± 0.07
Untreated	19.94 ± 0.24	8.94 ± 0.17	1.02 ± 0.04	0.68 ± 0.01	2.02 ± 0.10	1.89 ± 0.13	0.00 ± 0.00	0.00 ± 0.00
Overliming	18.76 ± 0.19	8.25 ± 0.59	0.87 ± 0.02	0.67 ± 0.00	2.24 ± 0.20	24.59 ± 0.16	9.82 ± 0.40	0.40 ± 0.01

^a^Total sugar consumption was calculated by the sum of the reduction of five sugars

^b^*C*_ethanol_ represents the ethanol production by 48 h

^c^*Y*_ethanol_ represents the ethanol yield at 48 h based on the total sugar consumption

### Effect of reactivated AC on detoxification

**Fig. 4:**
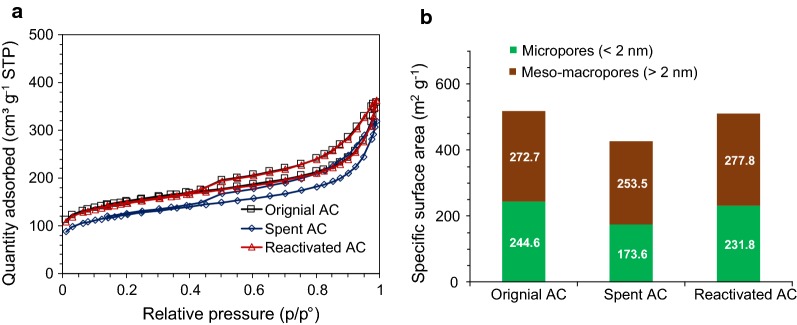
Isothermal plots (**a**) and surface areas of the original, spent and reactivated AC (**b**)

Surface area loss was observed for the spent AC after the detoxification treatment (Fig. 4). The Brunauer–Emmett–Teller (BET) surface area of spent AC was 427.1 m^2^ g^−1^, which presented a 17.4% decrease compared with the value of the original AC (517.3 m^2^ g^−1^). Additionally, the surface area of micropores (pore size < 2 nm) decreased from 244.6 to 173.6 m^2^ g^−1^, which was considered to be the key contribution of molecular adsorption. After thermal reactivation, the surface area could be improved to 98.7% (510.7 m^2^ g^−1^) of the original AC. The well recovered capability was achieved because the adsorbates (inhibitors) were volatized or thermal decomposed into gases and carbon during the thermal treatments.

**Fig. 5:**
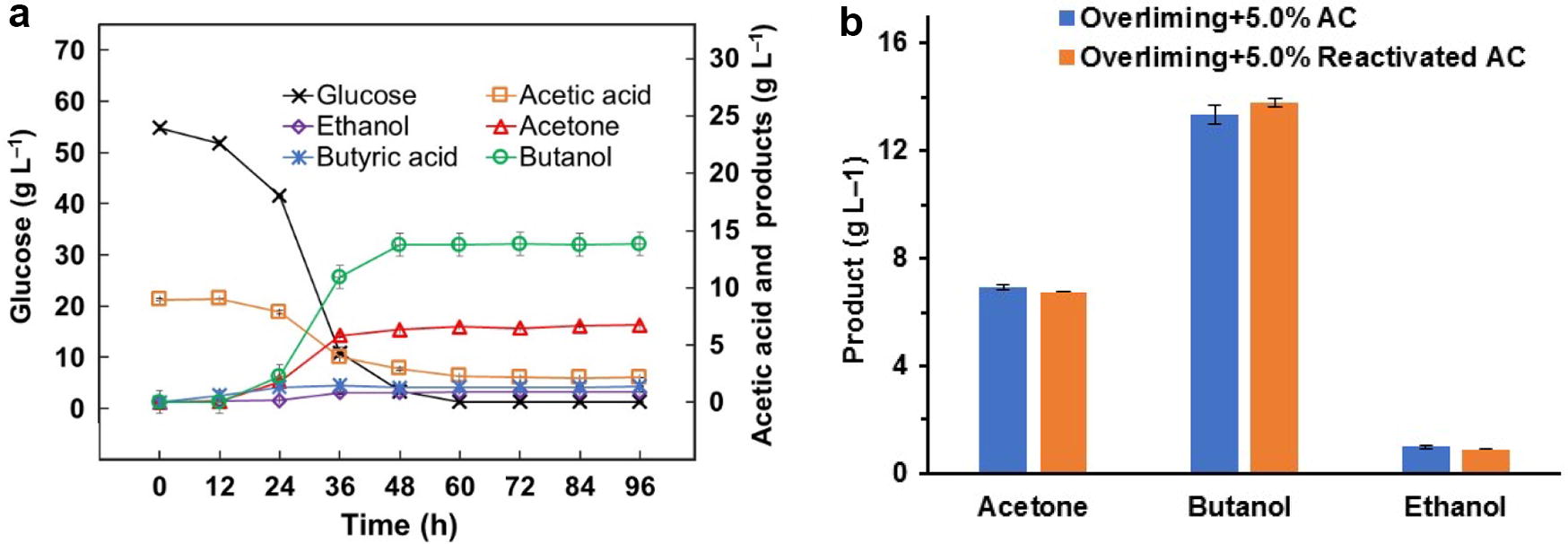
ABE fermentation of the prehydrolysate detoxified by sequential overliming and reactivated AC. **a** glucose consumption and ABE production, **b** ABE production comparison between original AC and reactivated AC detoxification

To examine whether the AC can be recycled in the sequential detoxification, the reactivated AC was used in a prehydrolysate detoxification for ABE fermentation. The results showed that the sugar loss in sequential overliming and reactivated AC was similar to that in sequential overliming and original AC (8.4% vs 8.0%). Approximately 94.2% of total sugars were consumed in 48 h and the final butanol concentration reached 13.81 g L^−1^. The butanol and ABE yield reached 0.22 and 0.34 g g^−1^ sugar, respectively (Fig. 5a). The results indicated that AC can be recycled in the sequential detoxification of biomass prehydrolysate for ABE fermentation (Fig. 5b).

## Conclusions

Overliming and AC alone could not make the prehydrolysates fermentable with *C. saccharobutylicum*. The sequential overliming and AC resulted in remarkable fermentability and high butanol yield at 0.22 g g^−1^ sugar, which was close to the pure glucose fermentation (0.25 g g^−1^ sugar). The synergistic effect of overliming and AC detoxification on ABE fermentation was perceived. It was observed that aliphatic acids were not changed with overliming and AC treatment. However, the overliming and AC showed significant difference in phenolic acid removal. Overliming removed much more 2,5-furandicarboxyaldehyde, 5-ethylfuran-2-carbaldehyde and 2,5-hexanedione compared to AC treatment, while AC treatment removed more phenolic acids than overliming. The difference in removing the dialdehydes/diketones and phenolic acids by overliming and AC could be the main reason for their synergic effect in detoxification of the prehydrolysates for ABE fermentation.

It was observed that AC was more effective in removing phenolic acids due to their hydrophobicity and overliming was more selective in removing certain dialdehydes and diketones due to the base-catalyzed aldo-condensation reactions. The effect of AC detoxification depended on the amount of AC used in the process. Yeast showed much more tolerance to the overliming treated prehydrolysate than *C. acetobutylicum.* The study suggested a different detoxification was needed for ABE fermentation of the biomass prehydrolysate as compared to ethanol fermentation.

## Methods

### Chemicals and microorganisms

Glucose, Reinforced Clostridial Medium (RCM), MgSO_4_, K_2_HPO_4_, KH_2_PO_4_, MnSO_4_·H_2_O, FeSO_4_·7H_2_O and agar were obtained from VWR (Radnor, PA). Granular activated carbon (20–40 mesh), Ca(OH)_2_, and CH_3_COONH_4_, were purchased from Alfa Aesar (Ward Hill, MA). NaCl and CaCO_3_ were obtained from Fisher Scientific (Fair Lawn, NJ). Biotin, H_2_SO_4_ (96%), HCl, *p*-aminobenzoic acid, thiamine, and yeast extract were acquired from Sigma Aldrich (St. Louis, MO). Peptone was purchased from Research Products International (Prospect, IL). Stock solutions (i.e., buffer, mineral, and vitamin) were prepared and stored at 4 °C. Buffer solution contained K_2_HPO_4_, KH_2_PO_4_, and CH_3_COONH_4_ at the concentration of 50, 50, and 220 g L^−1^, respectively. The mineral solution contained 40, 2, 2, and 2 g L^−1^ of MgSO_4_·7H_2_O, MnSO_4_·H_2_O, FeSO_4_·7H_2_O, and NaCl, respectively. Vitamin solution was composed of 1, 1, and 0.01 g L^−1^ of *p*-aminobenzoic acid, thiamine, and biotin, respectively.

The strain for ABE fermentation (*Clostridium saccharobutylicum* BAA-117) was acquired from the American Type Culture Collection. The spore suspensions were stored in sterilized deionized (DI) water at 4 °C. RCM medium (38 g L^−1^) was autoclaved (121 °C) for 15 min and then sparged with nitrogen to remove the oxygen. The spores were heat-shock (75 °C) for 10 min, inoculated to the sterilized RCM medium (50 mL), and then incubated (35 °C) in anaerobic chamber for 16–18 h. The optical density (OD_600_) was tested by UV–Vis spectrophotometer in terms of the absorption intensity at 600 nm to determine the cell concentration [39]. The cell was inoculated for fermentation with an OD_600_ value of 1.30.

The baker’s yeast *S. cerevisiae* (Fleischmann’s) was used for the ethanol fermentation. The yeast extract peptone dextrose medium (YPD), including glucose (20 g L^−1^), peptone (20 g L^−1^), yeast extract (10 g L^−1^), were prepared and sterilized. The yeast was pre-activated in YPD medium overnight, followed by washing with sterilized DI water. The OD_600_ were tested to determine the yeast concentration [30]. The initial inoculum concentration was 1.0 g L^−1^ for the yeast fermentation.

### Dilute acid pretreatment of biomass

Hybrid poplar (*Populus*) (0.25-in. grind) was provided by Idaho National Laboratory (Oregon, USA). The chemical composition of the untreated poplar was 49.42% glucan, 14.63% xylan, 0.80% galactan, 0.19% arabinan, 1.14% mannan, 26.69% lignin, 0.24% ash and 2.04% extractives. Before the hydrothermal reaction, 200 g of wood chips (dry weight) were soaked in 1.4 L aqueous 1.0% (*w/v*) sulfuric acid solution overnight. The pretreatment was conducted in a 2-L Parr batch reactor at 160 °C for 1 h. After cooling down to room temperature, the liquid fraction (prehydrolysate) from dilute sulfuric acid pretreatment was separated by vacuum filtration and stored at 4 °C.

### Detoxification of biomass prehydrolysates with Ca(OH)_2_ and activated carbon

Ca(OH)_2_ was added to adjust the pH of the prehydrolysates to 6. The glucose concentrations in the prehydrolysates were brought to approximately 65.75 g L^−1^ by adding around 55 g L^−1^ pure glucose. For the yeast fermentation, 10 g L^−1^ additional glucose was supplemented to make a final concentration of 20.75 g L^−1^. Overliming and AC treatments were performed to detoxify the prehydrolysates. All the detoxification treatments were conducted in duplicates.

Overliming method was used to detoxify the prehydrolysate [30]. Briefly, the pH value of the prehydrolysate (1 L) was brought to 10 with 15.32 g of Ca(OH)_2_. The prehydrolysate was then heated in a 60 °C water bath for 2 h. Subsequently, the pH of the prehydrolysate was adjusted to the desirable fermentation condition (pH = 6) using H_2_SO_4_. After removing the precipitates by centrifugation, the liquid phase was kept for GC/MS analysis and fermentation. The volume change after overliming treatment was negligible.

The AC treatment of the prehydrolysate (pH = 6) was conducted in a 250-mL shake flask with ground-glass stopper. After mixing 100 mL of prehydrolysate and 5.0% (*w/v*) of AC, the flask was placed on an orbital shaker (200 rpm) for 2 h. The prehydrolysate was collected after removing AC by vacuum filtration. DI water was supplemented to bring the prehydrolysate to the original volume.

The sequential overliming and AC method was performed for the detoxification of the prehydrolysate. Firstly, the prehydrolysate was detoxified by overliming method described previously. After removing the precipitates, the AC detoxification was performed in the overliming treated prehydrolysate. The adding amounts of AC were 1.0, 2.5, 5.0 and 10.0% (*w/v*), respectively, for the sequential overliming and AC detoxification.

### ABE and yeast fermentation

For each fermentation broth, the solution was adjusted to pH = 6 and filtered through the membrane syringe filter (0.22 µm) for sterilization. ABE fermentation was performed in a 125-mL serum bottle. The fermentation medium was composed of 45 mL of corresponding prehydrolysate (glucose concentration: ~ 60 g L^−1^), yeast extract (1 g L^−1^), 0.5 mL buffer, 0.25 mL mineral, and 50 μL vitamin solutions. CaCO_3_ powder (0.25 g) was added to maintain the pH during the fermentation process. After sparging with nitrogen for 10 min to purge the oxygen, the medium was immediately transferred into an anaerobic chamber. A 10% (*v*/*v*) growing bacteria was inoculated into the serum bottle to achieve a final volume of 50 mL. A rubber stopper and an aluminum sealing cap were used to seal the serum bottle. A needle (0.8 mm × 40 mm) was inserted into the rubber stopper to release the internal pressure. The bottle was incubated at 35 °C and samples (0.5 mL) were taken every 12 h until 96 h for HPLC analyses. The fermentation was conducted in duplicates.

Yeast fermentation was conducted in a 250-mL flask with 50 mL of the untreated or overliming-treated prehydrolysate. The glucose blank (20 g L^−1^) was prepared as reference fermentation. After inoculation of yeast, the flask was placed in an incubator shaker (150 rpm) at 30 °C. The samples (0.5 mL) were collected for HPLC analyses at 0, 1, 3, 6, 9, 12, 24 and 48 h, respectively.

### High-performance liquid chromatography (HPLC) analysis

Quantification of the fermentation products and short chain aliphatic acids were achieved with by an Agilent 2600 HPLC system (Detector: RID-10A; Column: Agilent Hi-Plex H). The mobile phase (aqueous 5 mM sulfuric acid solution) flow rate was at 0.6 mL min^−1^. The column temperature was kept at 45 °C throughout the sample run. The biomass sugars were quantified with a Bio-Rad HPX-87P column by the same HPLC system. The column temperature was maintained at 80 °C with DI water at the same flow rate for 35 min. According to the HPLC analysis, the prehydrolysates contained glucose (10.85 g L^−1^), xylose (8.93 g L^−1^), galactose (1.04 g L^−1^), arabinose (0.64 g L^−1^), mannose (1.94 g L^−1^), and sugar degradation compounds including formic acid (1.15 g L^−1^), acetic acid (6.08 g L^−1^), levulinic acid (1.12 g L^−1^), HMF (0.63 g L^−1^) and furfural (4.94 g L^−1^). The furfural concentration was higher than the result from GC/MS because of the loss from solvent extraction and nitrogen blowing. An Agilent 1290 LC-6540 connected to a quadrupole time-of-flight (Q-TOF) mass spectrometer was used to determine the phenolic acid removal. An Agilent Zorbax Eclipse Plus C18 column (4.6 × 100 mm, 3.5 µm particle size) was used to separate the phenolic acids in the prehydrolysate. The flow rate was controlled at 0.5 mL min^−1^ with two eluents: (A) H_2_O with 5 mM ammonium acetate and (B) acetonitrile with 5 mM ammonium acetate. The gradient started from 95% A during the first minute, then decreased to 5% A at 8 min and held for 1 min, and finally changed back to 95% A. The electrospray ionization (ESI) parameter was maintained as gas temperature (350 °C), gas flow (8 L min^−1^) and capillary voltage (3.5 kV) at negative mode. The analysis scan was from 50 to 500 *m/z*.

### Gas chromatography–mass spectrometry (GC/MS) analysis

To isolate the carbonyl inhibitors from the prehydrolysate, same amount of DCM (50 mL) was used to extract the compounds from 50 mL prehydrolysate twice. After going through anhydrous sodium sulfate (~ 10 g) column, the DCM-extracted solution was collected, and then concentrated to 5 mL by a nitrogen blowing concentrator (TurboVap II workstation). GC/MS analysis was performed on an Agilent 7890A GC and 7000 mass selective detector triple quadrupole. A DB-5 capillary column (J & W Scientific, 30 m length, 0.25 mm i.d., and 0.25 µm thickness) was employed to achieve chromatographic separation of the analytes. The electron ionization ion source was maintained at 250 °C and 70 eV. The mass spectra were scanned from 30 to 700. The oven temperature was held at 60 °C for 4 min to delay the solvents, and then increased to 105 °C (12 °C min^−1^ ramping, 2 min holding); to 160 °C (15 °C min^−1^ ramping, 1 min holding); to 250 °C (10 °C min^−1^ ramping, 2 min holding); and finally increased to 315 °C (10 °C min^−1^ ramping, 8 min holding). The accumulated running time was 40 min.

### Surface area analysis of activated carbon

The surface area of AC was determined by an ASAP 2020 surface area and porosity analyzer, using nitrogen absorption method. The ACs were vacuum degassed (250 °C) for 20 h before the analysis. Based on the isothermal plots obtained from the analysis, the BET total surface areas and t-plot micropore [< 2 nm, defined by IUPAC (International Union of Pure and Applied Chemists)] surface areas of the samples were obtained. The surface area of meso- and macropore (≥ 2 nm) was calculated by subtracting the corresponding micropore surface area from the BET total surface area.

### Reactivation process of activated carbon

The spent AC collected from detoxification process was washed by 1 M HCl aqueous solution. After being dried at 105 °C for approximate 20 h, the sample was transferred to a tube furnace, and reactivated at 650 °C for 2 h with the flow of nitrogen gas (10 cm^3^ min^−1^) [53]. After cooling down to room temperature, the surface area of the reactivated AC was determined. The efficiency of the detoxification using the reactivated AC was evaluated.

## Additional file

**Additional file 1: Figures S1–S46.** MS spectra of the tentatively identified compounds. **Figure S47.** Structures of the tentatively identified carbonyl inhibitors from the poplar prehydrolysate by GC/MS. **Figure S48.** UV–Vis spectra of the untreated poplar prehydrolysate, the prehydrolysates after overliming, 5.0% AC and sequential overliming with 1.0, 2.5, 5.0 and 10.0% AC. **Figure S49.** TIC-GC/MS chromatogram of the prehydrolysate detoxified with sequential overliming and 2.5% AC.

## Abbreviations

GC/MS: gas chromatography–mass spectrometry
AC: activated carbon
ABE: acetone–butanol–ethanol
HMF: hydroxymethylfurfural
DCM: dichloromethane
UV–Vis: ultraviolet–visible
BET: Brunauer–Emmett–Teller
OD: optical density
YPD: peptone dextrose medium
HPLC: high-performance liquid chromatography
Q-TOF: quadrupole time-of-flight
ESI: electrospray ionization
