# Supplementary material for: Effect of overliming and activated carbon detoxification on inhibitors removal and butanol fermentation of poplar prehydrolysates
Source: Biotechnol Biofuels. 2018 Jun 26;11:178. doi: 10.1186/s13068-018-1182-0 (PMC6020205; doi:10.1186/s13068-018-1182-0)
Supplement: Supplementary file 1 — Additional file 1: Figures S1–S46. MS spectra of the tentatively identified compounds. Figure S47. Structures of the tentatively identified carbonyl inhibitors from the poplar prehydrolysate by GC/MS. Figure S48. UV–Vis spectra of the untreated poplar prehydrolysate, the prehydrolysates after overliming, 5.0% AC and sequential overliming with 1.0, 2.5, 5.0 and 10.0% AC. Figure S49. TIC-GC/MS chromatogram of the prehydrolysate detoxified with sequential overliming and 2.5% AC. [file 13068_2018_1182_MOESM1_ESM.docx]

**Effect of overliming and activated carbon detoxification on inhibitors removal and butanol fermentation of poplar prehydrolysates**

Yu Zhang, Changlei Xia, Mingming Lu, Maobing Tu*

Department of Chemical and Environmental Engineering, University of Cincinnati, 2901 Woodside Drive, Cincinnati, OH 45221, United States

* Corresponding author

Email address: [tumg@uc.edu](mailto:tumg@uc.edu)

Tel: +1 513 556 2259

**Figure S1 – 46:** MS spectra of the tentatively identified compounds

**Figure S47:** Structures of the tentatively identified carbonyl inhibitors from the poplar prehydrolysate by GC/MS

**Figure S48:** UV-Vis spectra of the untreated poplar prehydrolysate, the prehydrolysates after overliming, 5.0% AC and sequential overliming with 1.0%, 2.5%, 5.0% and 10.0% AC

**Figure S49:** TIC-GC/MS chromatogram of the prehydrolysate detoxified with sequential overliming and 2.5% AC

**(b)**

**(a)**

**Fig. S1** MS spectra of furfural **(1)**: (a) Sample, (b) Standard.

**(a)**

**(b)**

**Fig. S2** MS spectra of 2-Acetylfuran **(2)**: (a) Sample, (b) Standard.

**(a)**

**(b)**

**Fig. S3** MS spectra of 2,5-Hexanedione **(3)**: (a) Sample, (b) Standard.


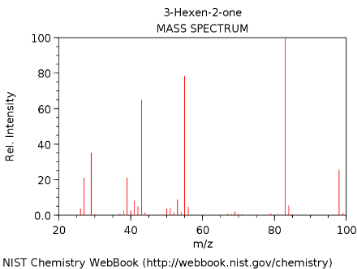


**(b)**

**(a)**

**Fig. S4** MS spectra of 3-Hexen-2-one **(4)**: (a) Sample, (b) Standard from National Institute of Standards and Technology (NIST).

**(b)**

**(a)**

**Fig. S5** MS spectra of 5-Methylfurfural **(5)**: (a) Sample, (b) Standard.

**(a)**

**(b)**

**Fig. S6** MS spectra of Phenol **(6)**: (a) Sample, (b) Standard.

**(b)**

**(a)**

**Fig. S7** MS spectra of Cyclotene **(7)**: (a) Sample, (b) Standard.

**(b)**

**(a)**

**Fig. S8** MS spectra of 2,5-Furandicarboxyaldehyde **(8)**: (a) Sample, (b) Standard.


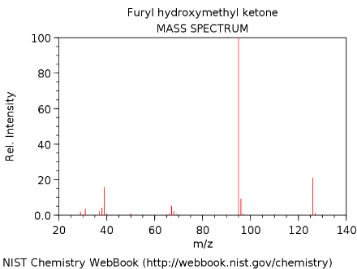


**(b)**

**(a)**

**Fig. S9** MS spectra of 2-Furyl hydroxymethyl ketone **(9)**: (a) Sample, (b) Standard from NIST.

**(b)**

**(a)**

**Fig. S10** MS spectra of 5-Ethylfuran-2-carbaldehyde **(10)**: (a) Sample, (b) Standard.

**(b)**

**(a)**

**Fig. S11** MS spectra of Benzoic acid **(11)**: (a) Sample, (b) Standard.

**(b)**

**(a)**

**Fig. S12** MS spectra of Hydroxymethylfurfural (HMF) **(12)**: (a) Sample, (b) Standard.

**(b)**

**(a)**

**Fig. S13** MS spectra of 3'-Methoxyacetophenone **(13)**: (a) Sample, (b) Standard.

**(b)**

**(a)**

**Fig. S14** MS spectra of 3,4,5-Trihydroxybenzaldehyde **(14)**: (a) Sample, (b) Standard.

**(b)**

**(a)**

**Fig. S15** MS spectra of Vanillin **(15)**: (a) Sample, (b) Standard.

**Fig. S16** MS spectra of Homovanillin **(16)** from the prehydrolysate sample.

**(b)**

**(a)**

**Fig. S17** MS spectra of Acetovanillone **(17)**: (a) Sample, (b) Standard.

**(b)**

**(a)**

**Fig. S18** MS spectra of Guaiacylacetone **(18)**: (a) Sample, (b) Standard.


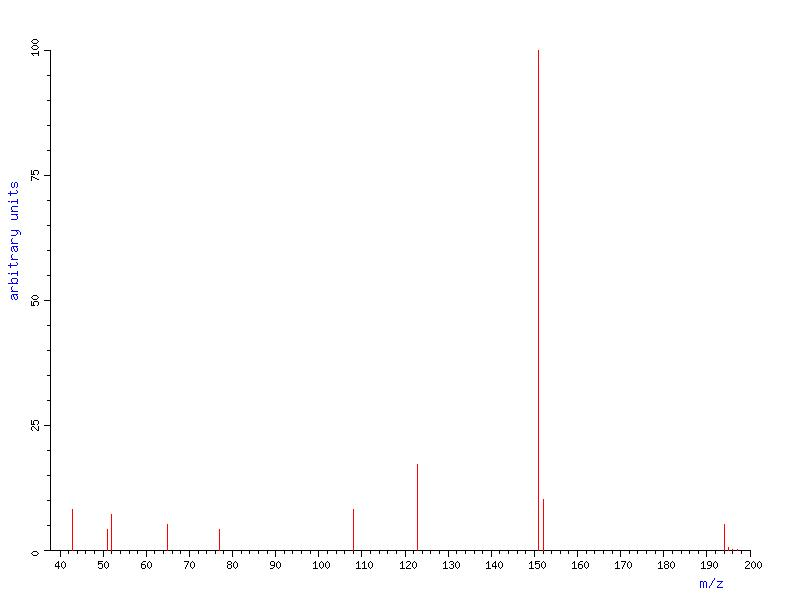


**(b)**

**(a)**

**Fig. S19** MS spectra of 1-(4-Hydroxy-3-methoxyphenyl)propane-1,2-dione **(19)**: (a) Sample, (b) Standard from Scifinder.

**Fig. S20** MS spectra of 1-(3,4,5-Trihydroxyphenyl)propane-1,2-dione **(20)** from the prehydrolysate sample.

**(b)**

**(a)**

**Fig. S21** MS spectra of Syringaldehyde **(21)**: (a) Sample, (b) Standard.

**Fig. S22** MS spectra of Hydroxypropiovanillone **(22)** from the prehydrolysate sample.

**(a)**


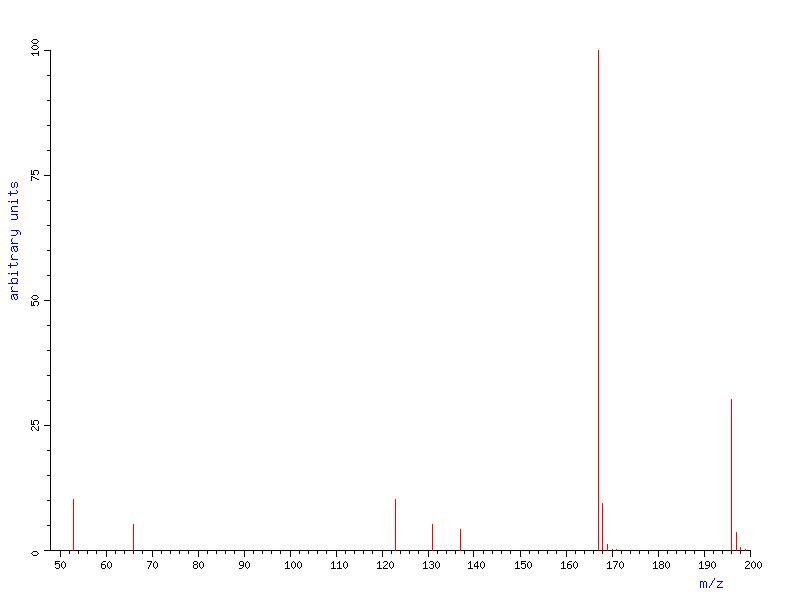


**(b)**

**Fig. S23** MS spectra of Homosyringaldehyde **(23)**: (a) Sample, (b) Standard from Scifinder.

***
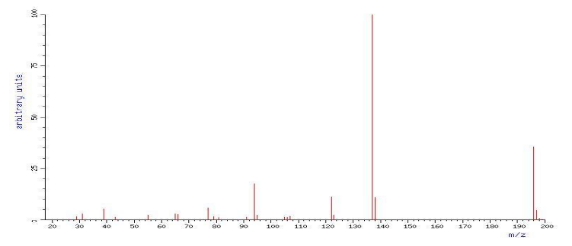
***

**(b)**

**(a)**

**Fig. S24** MS spectra of 1-Hydroxy-3-(4-hydroxy-3-methoxyphenyl)propan-2-one **(24)**: (a) Sample, (b) Standard from Scifinder.

 ****

**(b)**

**(a)**

**Fig. S25** MS spectra of Acetosyringone **(25)**: (a) Sample, (b) Standard.

**(a)**

**(b)**

**Fig. S26** MS spectra of Syringylacetone **(26)**: (a) Sample, (b) Standard.

**(a)**

**Fig. S27** MS spectra of 1-(4-Hydroxy-3-methoxyphenyl)-2-butanone **(27)** from the prehydrolysate sample.

**Fig. S28** MS spectra of 1-(4-Hydroxy-3,5-dimethoxyphenyl)propane-1,2-dione **(28)** from the prehydrolysate sample.

**Fig. S29** MS spectra of 1-Hydroxy-1-(4-hydroxy-3,5-dimethoxyphenyl)-2-propanone **(29)** from the prehydrolysate sample.

**Fig. S30** MS spectra of 2-Hydroxy-1-(4-hydroxy-3,5-dimethoxyphenyl)propan-1-one **(30)** from the prehydrolysate sample.

**Fig. S31** MS spectra of 2-Hydroxy-1-syringyl-ethanone **(31)** from the prehydrolysate sample.

**Fig. S32** MS spectra of 1-(3,4,5-Trimethoxyphenyl)-1,2-propanedione **(32)** from the prehydrolysate sample.

**Fig. S33** MS spectra of (4-Hydroxy-3,5-dimethoxybenzoyl)-acetaldehyde **(33)** from the prehydrolysate sample.

**Fig. S34** MS spectra of Gentisein **(34)** from the prehydrolysate sample.

**Fig. S35** MS spectra of 1-(4-Hydroxy-3,5-dimethoxyphenyl)pentane-1,2-dione **(35)** from the prehydrolysate sample.

**Fig. S36** MS spectra of 2-(4-hydroxy-3-methoxyphenyl)-1-(3,5-dihydroxyphenyl)ethanone **(36)** from the prehydrolysate sample.

**Fig. S37** MS spectra of 4-Hydroxyphenyl 3,4,5-trimethoxybenzoate **(37)** from the prehydrolysate sample.

**Fig. S38** MS spectra of 1,2-bis(4-hydroxy-3-methoxyphenyl)ethanone **(38)** from the prehydrolysate sample.

**Fig. S39** MS spectra of 1-(4-hydroxy-3,5-dimethoxyphenyl)-2-(4-hydroxy-3-methoxyphenyl)ethanon **(39)** from the prehydrolysate sample.

**Fig. S40** MS spectra of 2-(4-hydroxy-3,5-dimethoxyphenyl)-1-(4-hydroxy-3-methoxyphenyl)ethenone **(40)** from the prehydrolysate sample.

**Fig. S41** MS spectra of 2-Syringylacetosyringone **(41)** from the prehydrolysate sample.

**Fig. S42** MS spectra of Vanillosyringil **(42)** from the prehydrolysate sample.

**Fig. S43** MS spectra of 1,2-bis(4-hydroxy-3,5-dimethoxyphenyl)ethanone **(43)** from the prehydrolysate sample.

**Fig. S44** MS spectra of Syringil **(44)** from the prehydrolysate sample.

**Fig. S45** MS spectra of 1-(4-acetyl-3,5-dimethoxyphenyl)-2-(4-hydroxy-3,5-dimethoxyphenyl)ethane-1,2-dione **(45)** from the prehydrolysate sample.

**Fig. S46** MS spectra of Phenol, 4,4'-(1,2-ethanediyl)bis[2,6-dimethoxy-, diacetate **(46)** from the prehydrolysate sample.


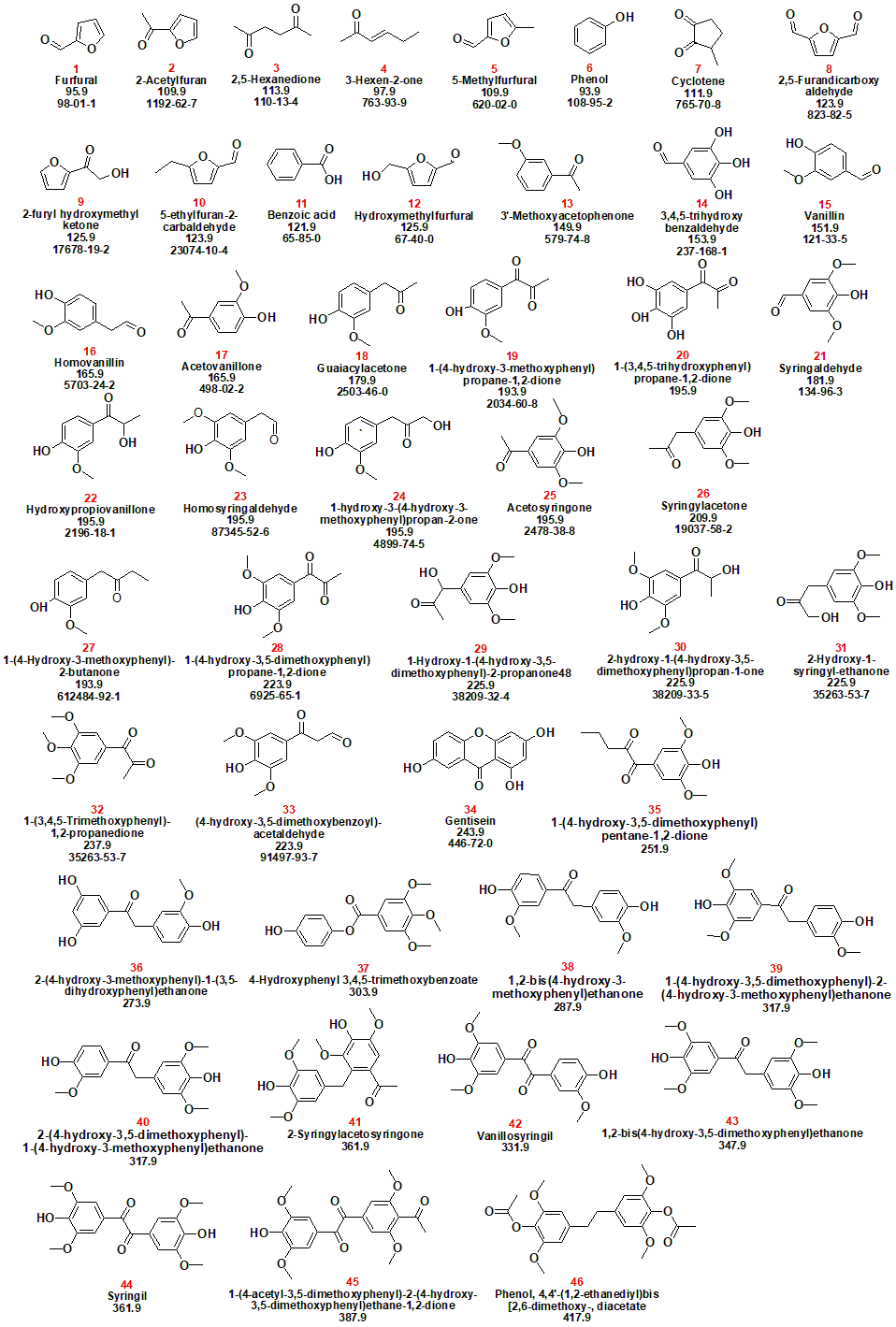


**Fig. S47** Structures of the identified carbonyl inhibitors from poplar prehydrolysate by GC/MS.

**Fig. S48** UV-Vis spectra of untreated poplar prehydrolysate, prehydrolysate after overliming, 5.0% AC and sequential overliming with 1.0%, 2.5%, 5.0% and 10.0% AC.

**Fig. S49** TIC-GC/MS chromatogram of the prehydrolysate detoxified with sequential overliming and 2.5% AC.
